# Acute Common Iliac Artery Occlusion Secondary to Blunt Abdominal Trauma From Motor Vehicle Accident

**DOI:** 10.7759/cureus.28271

**Published:** 2022-08-22

**Authors:** Khushboo K Agarwal, Mounika Gunduru, Moiuz Chaudhri, Eric Costanzo, Vistasp J Daruwalla

**Affiliations:** 1 Internal Medicine, Jersey Shore University Medical Center, Neptune City, USA; 2 Radiology, Yale University, New Haven, USA; 3 Radiology, Wayne State University Detroit Medical Center, Detroit, USA

**Keywords:** abdominal trauma, motor vehicle accident, complication, blunt trauma, acute common iliac artery occlusion

## Abstract

Blunt abdominal trauma infrequently leads to vascular injuries, and common iliac artery (CIA) injuries after motor vehicle accidents due to seat belt injury are very rare. Its posterior anatomic location and the pelvic bones usually protect the CIA. We describe a case of a young female presenting with acute blunt trauma to the abdomen after being a restrained driver in a motor vehicle accident and was found to have acute left CIA occlusion. The purpose of this case is to stress the importance of maintaining a high index of suspicion for vascular injuries in blunt abdominal trauma; we recommend early imaging diagnosis and timely treatment to mitigate its complications.

## Introduction

Common iliac artery (CIA) injuries after motor vehicle accidents are rare but require complex clinical management, leading to significant morbidity and mortality. Compared to penetrating iliac trauma, blunt iliac arterial injury is frequently associated with major pelvic fracture. Although rates of early hemorrhagic deaths are low, the incidence of septic complications increases with time [1]. CIA blunt trauma in the absence of pelvic bone fractures has been attributed to seat belts in motor vehicle accidents [2]. We present a rare case of a 54-year-old female who met with a motor vehicle accident leading to acute left CIA occlusion without pelvic bone fractures.

## Case presentation

A 54-year-old female presented to our hospital as a level 2 trauma resuscitation status after a motor vehicle accident. She was a restrained driver of a vehicle that rear-ended a parked car at high speed. There was significant damage to the front end of the patient's car, and airbags were deployed. The patient was found to be intoxicated on the scene of the accident. She sustained blunt head trauma with loss of consciousness. On arrival to the emergency department, the patient was somnolent but arousable. She reported that she had significant abdominal pain predominantly in the left lower quadrant. On examination, her breath sounds were clear and equal bilaterally with equal chest rise bilaterally. She had equal pulses in all four extremities. An abrasion was noted over the left lower abdomen along with small lacerations over the right hand. She demonstrated midline thoracic and lumbar spine tenderness to palpation without any focal neurological deficits.

**Figure 1:**
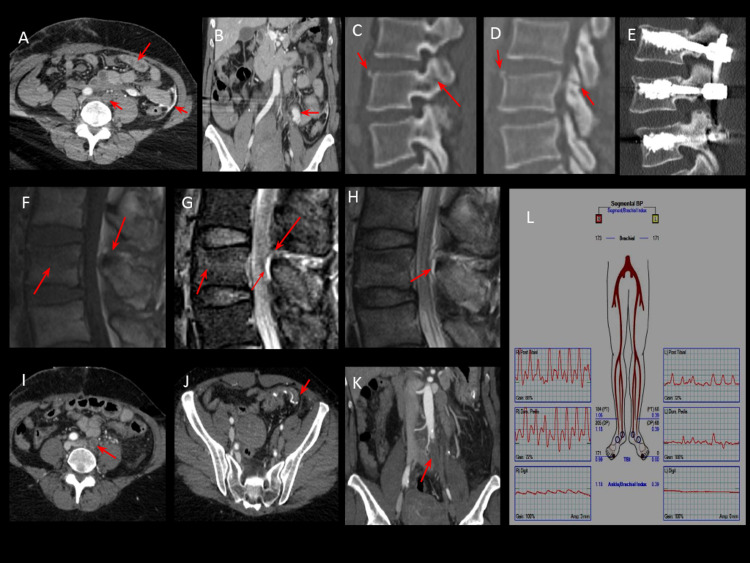
Acute occlusion of the left common iliac artery (A, B, I, J, and K) CT demonstrating occluded left common iliac artery, sigmoid colon injury with active extravasation, hemoperitoneum, and small bowel edema. (C, D, E) CT demonstrating unstable chance fracture status post-posterior percutaneous fusion with vertebroplasty. (F, G, H) MRI of the lumbar spine demonstrating unstable chance fracture with small epidural hematoma. (L) Ankle-brachial index of the patient demonstrating severely decreased ratio of 0.39 in the left posterior tibial artery and 0 in the dorsalis pedis artery, indicating severe arterial insufficiency compared to normal 1.1 on the right side.

A stat portable chest and pelvis radiograph was performed in the resuscitation room. Chest radiograph was negative for any acute intrathoracic process. Pelvic radiograph demonstrated no acute fracture or dislocation. The patient underwent a computed tomography (CT) scan of the head, abdomen, and cervical, thoracic, and lumbar spine. CT of the head was negative for any acute intracranial hemorrhage. The cervical and thoracic spine CT scan was negative for any acute fractures. CT scan of the abdomen demonstrated findings suggestive of sigmoid colon traumatic injury along with diffuse small bowel edema. The CT scan demonstrated active contrast extravasation in the left lower quadrant suggestive of active bleeding from traumatic mesenteric injury along with moderate amount of hemoperitoneum. Acute occlusion of the left CIA with reconstitution of the external and internal iliac arteries was also noted on the CT scan of the abdomen (Figures 1A, 1B, 1I, 1J, 1K). CT scan of the lumbar spine clearly delineated the acute nondisplaced fracture of the anterior superior corner and posterior superior facet of the L3 vertebrae suggestive of a chance unstable fracture (Figures 1C, 1D).

The patient became hypotensive in the emergency department, massive transfusion protocol was initiated, and the patient was urgently taken to the OR for surgical intervention. The patient required exploratory laparotomy with sigmoid resection stapled side-to-side functional and end-to-end antiperistaltic colorectal anastomosis.

MRI of the lumbar spine was performed for further evaluation, which demonstrated mild compression deformity of the L3 vertebrae along with superior articulating facet fractures and interspinous and ligamentum flavum disruption at L2-3. A small amount of blood products was also present within the posterior epidural space at L3 without significant mass effect. The patient underwent posterior percutaneous fusion of the L2 to L4 vertebrae with vertebroplasty of the L2, L3, and L4 vertrebraes. Post-fusion CT scan demonstrated vertebroplasty changes at L2, L3, and L4 with transpedicular screws in good alignment (Figure 1E).

Vascular surgery was consulted for the management of acute left CIA occlusion secondary to blunt trauma. The patient denied any left leg pain, numbness, tingling, or weakness. On physical examination, although the left lower extremity was colder than the right lower extremity, it demonstrated normal capillary refill without any visual discoloration and appeared well perfused. Clinically, the patient did not demonstrate any signs of acute limb ischemia. A bilateral lower extremity arterial duplex ultrasound scan was performed, which revealed a left ankle-brachial index of 0.39, indicating severe arterial insufficiency (Figure 1L). The right lower extremity was normal on clinical examination and arterial duplex study. The patient was initially started on heparin drip with 25,000 units of IV heparin infused at 750 units/hour.

The patient underwent left femoral cutdown and iliac thrombectomy. During the thrombectomy, a small filling defect was noted in the left CIA, which was interpreted as an intimal flap, and hence bilateral kissing common iliac arterial stents were placed. The patient later developed chest pain, and a CT angiography of the thorax and abdomen was performed, which demonstrated a large aortic arch dissection extending from the left subclavian artery to the bilateral common iliac stents. The aortic dissection from the aortic arch up to the diaphragm was thrombosed and was treated with an endovascular stent placement.

The patient’s chest pain has resolved but she still continues to have some difficulty in walking. Hence, she has been discharged to a rehabilitation service for further monitoring and physiotherapy.

## Discussion

Majority of the vascular injuries are due to penetrating trauma. Motor vehicle accidents leading to blunt trauma account for 7% to 9% of vascular injuries [2]. Seat belt syndrome has emerged as the new pattern of injuries due to widespread increase in the use of seat belts. CIA injuries have been attributed to seat belt trauma in the absence of pelvic bone fractures. Seat belt trauma also includes abdominal wall contusions, lumbar spine fractures or subluxations, CIA, or aortic vascular injuries [2]. Our case highlights the traumatic injuries expected in a case of a restrained driver in a high-speed motor vehicle accident and demonstrates the importance of looking out for typical seat belt injuries in these cases. Deep vascular injuries secondary to seat belts are very rare, with the aorta and the inferior vena cava being the most frequently injured vascular structures. The incidence of iliac artery injuries from trauma ranged from 0.4% to 7.1% [3]. Currently, two combined mechanisms have been proposed to explain the pathophysiology of deep vascular injuries. The force related to blunt abdominal trauma, known as compression force, is produced by seat belt traction on the muscle wall and viscera between the belt and the spine. Compression force leads to a sudden increase in intraperitoneal pressure that may cause the avulsion of small branches from their origin. Tearing may lead to slow peritoneal hemorrhaging, further delaying diagnosis. The “deceleration force” is considered to be the cause of intimal tears with secondary intravascular thrombosis, which may even occlude the arterial lumen. It is generally accepted that the association of these two different types of force may create other shearing forces provoking vascular wall discontinuity or an intimal flap. The bony pelvis and the posterior position of the aorto-iliac system protect the CIA to a large extent, but the same shearing deceleration injuries may cause intimal tears [4].

CIA injuries are usually diagnosed and evaluated based on clinical suspicion such as the presence of pulse deficit, bruit, expanding hematoma, arterial bleeding, poor capillary filling, and cold extremities. Advancement in imaging modalities has played a critical role in early diagnosis and management of these vascular injuries. Contrast-enhanced CT scan performed for our patient not only delineated the colonic and spinal injuries but also assisted in the timely diagnosis of the left CIA occlusion. Symptoms of vascular injuries are commonly delayed but may progress to severe outcomes such as acute limb ischemia; hence, timely diagnosis and management of vascular injuries is critical in such cases. Acute limb ischemia is a rare clinical presentation for blunt aorto-iliac arterial injuries, and most patients are initially asymptomatic with a median delay in operative repair of 15 days (range: 2 days to 36 years) [5,6]. CIA vascular territory injuries are strongly associated with lower extremity amputations, which lead to major morbidity for the patients. Therefore, timely intervention is critical. Since most amputations occur after a revascularization procedure has been performed, subsequent amputation in this group is dependent on these concurrent injuries. In patients with a myriad of amputation risk factors, i.e., high-grade pelvic fractures and soft tissue injuries, and lower extremity trauma, early amputation along with vascular control may be beneficial [3]. All injuries reported by Tuech et al. were treated with conventional operative bypass grafting. Resection and direct end-to-end anastomosis are performed after mobilization of the hypogastric artery. Due to inherent challenges of hypogastric artery division to gain more length, vein grafts have provided satisfactory results. Although promising in the short term, difficulties associated with intimal flap transversion along with the young age of the patients support a cautionary approach in exercising percutaneous treatment with stems. Regardless, surgical intervention appears to produce excellent long-term hemodynamic and clinical results [3,7]. Advances and increasing implementation of endovascular capabilities for vascular trauma patients with CIA injuries may benefit from endovascular treatment. In future, endovascular treatment will likely play a vital and time-sensitive role in the treatment of vascular injuries alone or in conjunction with open surgical techniques. Endovascular techniques may hopefully improve the rates of limb amputations and decrease the procedural morbidity often seen with surgical techniques in these cases.

## Conclusions

Acute CIA occlusion is a rare vascular injury associated with blunt abdominal trauma secondary to a motor vehicle accident of a restrained passenger. Seat belt injuries are most likely due to shearing forces generated during rapid deceleration leading to an intimal flap, which leads to thrombosis of the vessel. These injuries are associated with rapid lower extremity amputation and death even after aggressive surgical revascularization. Although surgical revascularization appears to be the main choice of treatment, endovascular techniques may have an increasing role in the future. Through this case, we not only stress the importance of maintaining a high index of suspicion for CIA vascular injuries in patients with blunt abdominal trauma but also delineate the significance of early imaging diagnosis and aggressive treatment to mitigate its complications.
